# Reexamination of the Species Assignment of *Diacavolinia* Pteropods Using DNA Barcoding

**DOI:** 10.1371/journal.pone.0053889

**Published:** 2013-01-15

**Authors:** Amy E. Maas, Leocadio Blanco-Bercial, Gareth L. Lawson

**Affiliations:** 1 Woods Hole Oceanographic Institution, Woods Hole, Massachusetts, United States of America; 2 Department of Marine Sciences, University of Connecticut, Groton, Connecticut, United States of America; University of Canterbury, New Zealand

## Abstract

Thecosome pteropods (Mollusca, Gastropoda) are an ecologically important, diverse, and ubiquitous group of holoplanktonic animals that are the focus of intense research interest due to their external aragonite shell and vulnerability to ocean acidification. Characterizing the response of these animals to low pH and other environmental stressors has been hampered by continued uncertainty in their taxonomic identification. An example of this confusion in species assignment is found in the genus *Diacavolinia*. All members of this genus were originally indentified as a single species, *Cavolinia longirostris*, but over the past fifty years the taxonomy has been revisited multiple times; currently the genus comprises 22 different species. This study examines five species of *Diacavolinia*, including four sampled in the Northeast Atlantic (78 individuals) and one from the Eastern tropical North Pacific (15 individuals). *Diacavolina* were identified to species based on morphological characteristics according to the current taxonomy, photographed, and then used to determine the sequence of the “DNA barcoding” region of the cytochrome *c* oxidase subunit I (COI). Specimens from the Atlantic, despite distinct differences in shell morphology, showed polyphyly and a genetic divergence of <3% (K2P distance) whereas the Pacific and Atlantic samples were more distant (∼19%). Comparisons of *Diacavolinia* spp. with other *Cavolinia* spp. reveal larger distances (∼24%). These results indicate that specimens from the Atlantic comprise a single monophyletic species and suggest possible species-level divergence between Atlantic and Pacific populations. The findings support the maintenance of *Diacavolinia* as a separate genus, yet emphasize the inadequacy of our current taxonomic understanding of pteropods. They highlight the need for accurate species identifications to support estimates of biodiversity, range extent and natural exposure of these planktonic calcifiers to environmental variability; furthermore, the apparent variation of the pteropods shell may have implications for our understanding of the species’ sensitivity to ocean acidification.

## Introduction

Thecosome pteropods (Mollusca, Gastropoda) are a group of holoplanktonic animals that have been the focus of recent research because of their susceptibility to ocean acidification [1]. Bearing thin aragonite shells, the calcification of these animals has been shown to be reduced when they are exposed to the projected changes in surface water pH and saturation state of the future ocean [2], [3], [4]. Studies of metabolism, however, reveal a complex sensitivity to pH dependent upon natural exposure and synergistic stressors [5], [6], [7]. Understanding the response of these animals to ocean acidification, and understanding the broader ecosystem impacts of their sensitivity, has been slowed by a serious lack of information about the ecology, physiology and ecosystem function of pteropods. Although the ecological importance of pteropods is not clearly established, it is known that pteropods can become a numerically dominant member of the zooplankton community in temperate and polar seas [8] and it has been hypothesized that the species *Limacina inflata* is the most abundant gastropod in the world [9]. Growing research suggests that pteropods can be significant consumers of primary production [10], [11], [12], substantially contribute to carbon flux [13], [14], [15], [16], and serve as a key food item for a number of species including other zooplankton, fish, seabirds and whales [8], [9], [17], [18], [19], [20], [21].

Our expanding understanding of the importance of pteropods in food webs and biogeochemical cycling is complicated by a lack of clarity in the taxonomic literature for this group. As the ocean environment changes as a result of the complex interaction between rising temperature, changing O_2_ availability, shoaling of the saturation horizon for calcium carbonate compounds, as well as differences in water pH, it becomes more important to differentiate between distinct species in order to understand functional variability and resilience at population levels. Furthermore, in conjunction with generation time, high levels of genetic variability within a population may differentiate between species or populations that survive and those which are lost as the environment changes due to anthropogenic forcing [22], [23], [24]. The distribution and diversity of pteropod species, however, remains in question.

Pteropods, consisting of the two orders Thecosomata (shelled) and Gymnosomata (naked), have had a tumultuous systematic history starting in the 1800’s [9]. Originally grouped in the class of Pteropoda, only recently have they been regrouped with opisthobranchs and reunited as monophyletic sister taxa under the Gastropod class of Opisthobranchia using modern molecular techniques [25], [26]. The early taxonomic work on thecosomes was dependent upon net tow samples of formalin-preserved, frequently unbuffered specimens, and the recent traditional classification is marked by a high degree of “splitting” into species, subspecies, and formae [27]. Preserved thecosomes are frequently difficult to identify, as many of the delicate external morphological features diagnostic of some families, such as the gelatinous pseudoconch and the long external mantle appendages, are never seen in a natural conformation in net-captured animals [28]. Consequently, taxonomists have traditionally used the less delicate aragonitic shell to classify and identify organisms, despite the tendency of this structure to dissolve in acidic preservation techniques. This classical shell-based morphological taxonomy described a group which consists of a number of cosmopolitan or bipolar species found in multiple ocean basins, with variations in shell shape, size and color that transition over latitudinal or ocean basin scales and that have often been designated as sub-specific formae [9], [27]. Over time it has become clear that reliance solely upon shell morphology is an inadequate way of describing inter- and intra-species level variation in pteropods, and a number of designations have been reassessed. For example, populations of *Limacina helicina*, one of the most abundant pteropods and the dominant thecosome in polar regions, were originally believed to be the same species in the Arctic and Antarctic, but have recently been shown through genetic, behavioral, and physiological factors to be separate at a species level [29]. Furthermore, work spanning the various pteropod families indicates that although some circumglobal distributions and forma gradations are supported by genetic evidence, others are not, suggesting the need for closer examinations of the taxonomic classification of several pteropod genera [26].

The genus *Diacavolinia* (Order Thecosomata, Suborder Euthecosomata, Family Cavoliniidae, Subfamily Cavoliniinae) [30], [31], is one of the most heavily radiated groups, and is the most recently established taxonomic classification within the pteropods. Of the shelled pteropods, the Cavoliniinae are thought to be the most derived, having developed gills and the most complex mantle structures of the thecosomes, theoretically to provide greater surface area and stability in the planktonic environment [32], [33]. The *Diacavolinia* are a common and sometimes very abundant genus of subtropical thecosomes which were originally classified as one species, *Cavolinia longirostris* [34]. As a result of the findings of Tesch, during the Dana expeditions, this species was later re-classified as having three formae: *longirostris, strangulata*, and *angulata/angulosa* [31], [35], [36]. From these expeditions, which sampled extensively in Indo-Malayan waters and more sparsely from 20° N –40° S in the Atlantic Ocean, the first biogeography of this group was established with *strangulata* and *angulata/angulosa* absent from the Atlantic, and the Atlantic forma, *longirostris*, occasionally found in Indo-Pacific samples. The variability in shell morphology within these formae is mentioned by Tesch who described the *Cavolinia longirostris angulata* forma of the Pacific voyage as having a characteristic bifid rostrum, although noting that the rostrum was sometimes completely absent from similarly-sized specimens [36]. In Atlantic specimens Tesch notes that there was quite a range in animal size and that in smaller animals the rostrum, as well as the side-processes (spines), were significantly less developed [35].

In the early 1970’s van der Spoel re-examined the species *Cavolinia longirostris,* increased the number of forma to six, adding *limbata, flexipes* and *mcgowani,* and suggested that there were more clear basin level differences in their distribution [37], [38], [39]. The formae *longirostris* and *limbata* were assigned cosmopolitan distribution, *strangulata* was documented as inhabiting the Atlantic and Pacific basins, *angulosa* was found in the Indo-Pacific, *flexipes* in the Red Sea, and *mcgowani* exclusively in the Pacific Ocean [39]. In contrast to McGowan [40] who felt that variations in shell type were dependent upon variation in environmental conditions and a high degree of phenotypic plasticity, van der Spoel cited the consistency of distributional patterns and an unclear mechanism for the genesis of the distinct shell angles and characteristics to justify the naming of multiple formae. In time the species *Cavolinia longirostris* was raised to genus level, and renamed as *Diacavolinia longirostris* [30], based on the fact that individuals of the species lose their juvenile protoconch-I and –II in a different way from all other members of the Cavoliniinae subfamily. Finally in 1993, after an extensive review of museum specimens, van der Spoel and colleagues reorganized the genus and elevated the formae to species, after which it contained 22 species, one of which retained 2 formae. He argued that separate species were necessary based on the fact that there were no geographic patterns in their variation and no zones of transition; although he noted that sympatry of these different species was very common, he stated that individuals could accurately be identified in mixed samples.

These arguments, particularly the extreme frequency of sympatry, raise the question of what form of niche partitioning or mechanism of evolutionary differentiation could lead to such a variety of closely-related species coexisting in the plankton. Van der Spoel and his colleagues suggested that an improved separation of the male and female parts and the presence of a closed gonoduct may have produced rapid radiation of the genus [31]. Furthermore, since this group undergoes rapid growth of the adult teloconch shell followed only by minor additions of shell along the aperture and spines, van der Spoel et al. [31] asserted that co-occurrence of different shell sizes and shapes could not be a product of ontogenetic variation since the shapes cannot be explained through linear growth. Disentangling the identification of these species and the importance of ontogeny is made complicated, however, by the facts that the key established by van der Spoel et al. [31] is based exclusively on slight variations in shell shape and can only be used on adult animals. If valid, the apparent high degree of diversity in this genus could be associated with interesting physiological and ecological differences between co-occurring and recently separated species, meriting further investigation. Overall, due to the complexity of the species assignments of this group, *Diacavolinia* may benefit from reanalysis with the addition of modern molecular taxonomic techniques.

One of the approaches to address such questions is the use of DNA barcoding [41]. This technique enables the recognition and discrimination of an individual’s species based on a conserved short DNA sequence present in all genomes; the most commonly accepted for metazoans being a fragment from the gene for the mitochondrial cytochrome *c* oxidase subunit I [41]. DNA barcoding has been applied to a wide range of marine metazoans [42], including pteropods [26], [43]. Although subject to confusion and controversy about its use as a taxonomic instrument *per se* [44], [45], [46], this technique has proven to be a powerful tool for addressing and resolving systematic problems, when combined with traditional morphological, ecological, and physiological methods [47]. DNA barcoding is especially useful when morphological discrimination is difficult or impossible (e.g., early life stages, subtle or lacking diagnostic characters, cryptic species).

The objective of this study is to reexamine the species assignment of *Diacavolinia* pteropods using DNA barcoding. Individuals sampled in the Atlantic and Pacific Oceans were identified to species following the morphology-based key of van der Spoel et al. [31], and then genetically analyzed at the cytochrome *c* oxidase subunit I barcoding region. Additional insight was also gained by comparing individuals of the *Diacavolinia* genus to species from the closely-related *Cavolinia* genus. The resulting findings about biodiversity and population level variability were considered in light of their implications for pteropod response to environmental stressors, such as ocean acidification.

## Methods

### Specimen Collection, Preservation, and Morphological Analysis

Individuals collected for morphological and later genetic analysis were sampled during cruise OC 473 on the *RV Oceanus* (August 2011) in the Northwest Atlantic. Individuals from the eastern tropical North Pacific, obtained aboard the *RV Seward Johnson* (October and November 2007) were also analyzed. Samples from the Northwest Atlantic were captured using either a 1 m diameter, 150 µm mesh Reeve net towed through the upper 100 m at night or vertically stratified casts of a 1 m^2^ Multiple Opening/Closing net and Environmental Sensing System (MOCNESS) equipped with 150 µm mesh nets [48]. Pacific samples were collected with a 61 cm-diameter 335 µm-mesh bongo net trawl down to 100 m at night. Latitude and longitude coordinates are available for all specimens (Table 1).

**Table 1:** Information for the *Diacavolinia* specimens collected, analyzed and identified for this study (by A. Maas), as well as other *Cavolinia spp.* sequences retrieved from GenBank.

Species	Basin	Sample	N	Location	GenBank	Notes on classification
*D. robusta*	Atlantic	Ga32.9	2	38.50 N 52.00 W	JX183560	Hump present
		Ga32.11	2	38.51 N 51.96 W	to	No constriction in rostrum
					JX183563	No notch in rostrum
						Small spines, hooked
*D. strangulata*	Atlantic	Ga32.9	3	38.50 N 52.00 W	JX183564	Hump present
		Ga32.11	12	38.51 N 51.96 W	to	Constriction present
		Ga32.15	8	40.93 N 52.07 W	JX183586	Spines not bent dorsally
*Diacavolinia* spp.	Atlantic	Ga32.9	7	38.50 N 52.00 W	JX183587	No hump
juvenile		Ga32.15	4	40.93 N 52.07 W	to	No constriction in rostrum
					JX183597	No notch in rostrum
						Lateral spines absent
*D. atlantica*	Atlantic	Ga32.9	4	38.50 N 52.00 W	JX183598	No hump
					to	Notch present
					JX183601	Constriction present
						Wide aperture
						Shell >8 mm
*D. elegans*	Atlantic	Ga32.9	11	38.50 N 52.00 W	JX183602	No hump
					to	No notch
					JX183612	No lateral elongation of spines
						Spines bent slightly dorsally
						Lip shoulders small
*D. vanutrechti*	Pacific	Ga32.16	3	13.01 N 105.01 W	JX183614	No hump
					to	No notch
					JX183616	No lateral elongation of spines
						Spines bent slightly dorsally
						Lip shoulders larger
*C. gibbosa*	Atlantic	Ga05.1.1		33.52 N 69.96 W	FJ876856	[26]
*C. globulosa*	Atlantic	Ga43.3.1		14 N 55 W	FJ876857	[26]
*C. longirostris*	Atlantic	Ga32.1.1		24.95 N 60.53 W	FJ876859	[26]
	Atlantic	Ga32.1.2		24.95 N 60.53 W	FJ876860	[26]
*C. tridentata*	Indian	Ga57.1.1		46.35 S 140.54 E	FJ876861	[26]
*C. uncinata*	Indian	Ga29.6.2		17.47 S 121.43 E	FJ876864	[26]
	Indian	Ga29.7.1		16.23 S 119.62 E	FJ876865	[26]
	Atlantic	Ga29.9.1		11.38 N 20.35 W	FJ876866	[26]
	Atlantic	Ga68.2.3		11.38 N 20.35 W	FJ876858	[26]
	Atlantic	Ga29.1.1		24.95 N 6 0.53 W	FJ876862	[26]
	Atlantic	Ga29.9.2		14 N 55 W	FJ876863	[26]
	Atlantic	Ga82.3.1		13.42 S 0.65 W	FJ876867	[26]
	Atlantic	Ga82.3.2		13.42 S 0.65 W	FJ876868	[26]

Notes describe the taxonomic characters used to make the morphological classification for this analysis based on the 1993 van der Spoel key.

After collection, samples were preserved in 95% ethanol which was changed once after ∼24 h. All Atlantic samples were stored at –20°C to prevent DNA degradation; Pacific samples were not kept at a controlled temperature. In the lab, specimens were microscopically identified to species using the *Diacavolinia* key in van der Spoel et al. [31]. Photographs were taken of each *Diacavolinia* species to provide later comparison. When possible, shells were removed prior to DNA extraction to avoid both the possible pH interference due to the calcium carbonate and contamination from other taxa, since the presence of some other planktonic components (mostly ostracods and copepods) inside the shell chambers was common.

### DNA Extraction Procedure Tests

A series of initial tests was required to establish a reliable protocol for extraction and purification of the pteropod DNA. Three DNA purification/sample preparation kits were tested on whole individuals. Initially, extractions were carried out using DNeasy® Blood & Tissue Kit (Qiagen), with an elution volume of 200 µL in AE buffer, following the conditions established by the manufacturer with an initial digestion time of 2 h. After a number of unsuccessful PCR attempts for conserved regions on the genes COI [49], Cyt *b* [50] and 18S [51], 100 µL aliquots of five of the previous extractions were run through the *OneStep™* PCR Inhibitor Removal Kit (Zymo Research Corp.), a column matrix “designed for efficient removal of polyphenolic compounds, humic/fulvic acids, tannins, melanin, etc” according to the manufacturer product information sheet. Finally, the E.Z.N.A.® Mollusc DNA Kit (Omega Bio-Tek Inc.) was applied to both new individuals and to 50 µL aliquots from the DNeasy® extractions. All E.Z.N.A.® final extractions and re-extractions were carried out following the manufacturer recommendations, with lysis incubations at 60°C for ∼3 h, and a final elution on 200 µL independent of the body size of the animal.

After extraction, PCR amplification reactions were performed in a total volume of 25 µL, including 5 µL of 5× Green GoTaq® Flexi Buffer, 2.5 µL of 25 mM MgCl_2_, 1 µL of dNTPs (final concentration 0.2 mM each), 1 µL of each primer (10 µM), 0.75 units of GoTaq® Flexi DNA Polymerase (Promega) and 2 µL of DNA sample. PCR cycle conditions were relaxed, with annealing temperatures of 45°C/50°C/52°C for COI/Cyt *b*/18 S respectively. PCR products were checked by electrophoresis on a 1% agarose/TBE gel. Crossed PCR reactions on mixes of different extractions were carried out to confirm the presence of PCR inhibitors after some of the protocols.

As a result of findings from the above trials, all individuals were subsequently extracted using the E.Z.N.A.® Mollusc DNA Kit (OMEGA), following the manufacturer recommendations, with lysis incubations at 60°C for ∼3 h, and a final elution on 200 µL independent of the body size of the animal.

### DNA Sequencing

After extraction, PCR amplification reactions for the barcoding region were performed, using M13-tailed [52] LCO1490 and HCO2198 universal primers for COI [49]. The PCR protocol included an initial denaturation step of 94°C (3 min), followed by 35 cycles of denaturation at 94°C for 35 s, annealing at 45°C for 45 s, and extension at 69°C for 45 s. A final extension phase at 69°C for 15 min was followed by storage at 4°C. PCR products were checked by electrophoresis on a 1% agarose/TBE gel. PCR products were excised from the gel and purified with QIAquick Gel Extraction Kit (Qiagen). The purified PCR products were sequenced using the tailed M13 tails (both forward and reverse) and Big Dye Terminator Ver. 3.1 kit (Applied Biosystems Inc., ABI), and run on an ABI 3130 Genetic Analyzer capillary DNA sequencer. All sequences were deposited in GenBank (accession numbers JX183560-JX183616).

### Statistical Analysis

For comparison, additional sequences for certain *Cavolinia* species from Jennings et al. [26] were retrieved from GenBank (Table 1). No photographs are available for these samples. Note that the Jennings et al. samples include two specimens identified as *Cavolinia longirostris* from the Atlantic. This is an outdated identification, however, since as described above, this species has been split into a number of species and elevated to its own genus, *Diacavolinia*. The Jennings et al. *C. longirostris* is thus presumably some undetermined species of *Diacavolinia*.

Sequences were edited using MEGA ver. 5 [53], and aligned using the ClustalW [54] as implemented in MEGA. Parsimony haplotype networks (gene genealogies) were constructed with TCS Ver. 1.2.1 [55], in order to visualize the diversity and phylogenetic relationships among the different haplotypes and provide qualitative assessment of their distributions across the different individuals from the Atlantic Ocean. Haplotype diversity and Tajima’s *D* neutrality test were calculated with DnaSP ver. 5 [56].

To investigate intra-specific, inter-specific, and inter-generic relationships for *Diacavolinia* and *Cavolinia*, genetic distances were obtained following the Kimura 2-parameter (K2P) method [57], since this method has become the metric most widely used in barcoding studies [26], [42], [58]. To study the differences on the distances a) within the *Cavolinia* genus (between species, excluding *Diacavolinia*), b) between the *Cavolinia* and *Diacavolinia*, and c) between the Atlantic and Pacific *Diacavolinia*, Mann-Whitney *U* tests were carried out. To reduce the probability of false positives, strict Bonferroni correction [59] was applied for the multiple comparisons (α_corrected_ = 0.017). Although there is a lack of independence among the measured genetic distances, this method has frequently been used to explore differences between taxonomic levels [58].

To investigate the species assignment by a monophyly criterion, a tree analysis was conducted. Trees were constructed based on Maximum Likelihood (ML) and Bayesian Inference (BI), and were carried out with RAxML Ver. 7.2.3 [60], [61] and MrBayes Ver. 3.2 [62] respectively. ML analysis was run under the GTR model of nucleotide substitution with the Γ model of rate heterogeneity (GTRGAMMA) and a completely random starting tree. Bootstrap support for the nodes was obtained after 10,000 replicates. BI analysis was run for 3,000,000 generations, with a sample frequency of 1000 generations. The first 500 trees were discarded as burn-in, so 2500 trees were accepted from each run. Clade support on the nodes of the trees corresponds to their Bayesian Posterior Probability (BPP), and was considered significant only when BPP>0.90.

### Ethics Statement

Individuals were collected from international waters, exempting them from legislation; however, all scientific shipboard activities were conducted in such a way as to comply with the current laws of the United States of America. The study organisms are invertebrates, and as such no restrictions apply to their handling as experimental organisms.

## Results

### Species Identification Based on Morphology

**Figure 1:**
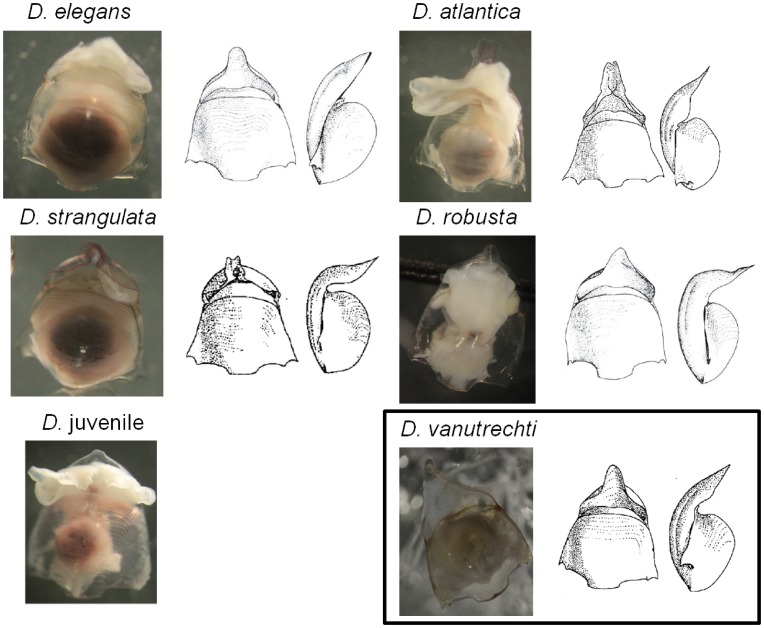
*Diacavolinia* species. Photographs of the various shell morphologies from the Atlantic and Pacific (black box) samples and drawings of the holotypes reproduced from van der Spoel et al. [31]. All photographs are from the ventral view except for *D. robusta* which is depicted from the dorsal view to show the rostral curvature. Drawings are of the ventral (left) and side (right) view of empty shells. Note that the photographs are not to scale.

Using current references and accepted morphologically-based species descriptions [31], we identified four species of *Diacavolinia* from the Atlantic Ocean (72 morphologically examined and 53 successful DNA extractions of 54 attempts) and one species in the Pacific Ocean (15 morphologically examined and 3 successful DNA extractions out of 10 attempts). We documented four species from a single Reeve net tow to 100 m in the Northwest Atlantic (Table 1, Figure 1). One additional group of individuals may not have completed the transition to adult shell morphology so it has also been classified as a possible juvenile shell form. Two other sites from the same cruise collected with the MOCNESS had a similar species composition, but lower densities and diversity. It was impossible to examine the morphological features of the individuals whose sequences were taken from GenBank, and as a result the Jennings et al. individuals [26] could not be identified to species level and were left as *Cavolinia longirostris* in all further documentation and treated as *Diacavolinia* in our comparative analyses.

### Molecular Analysis: DNA Extraction, PCR, and Sequencing

**Figure 2:**
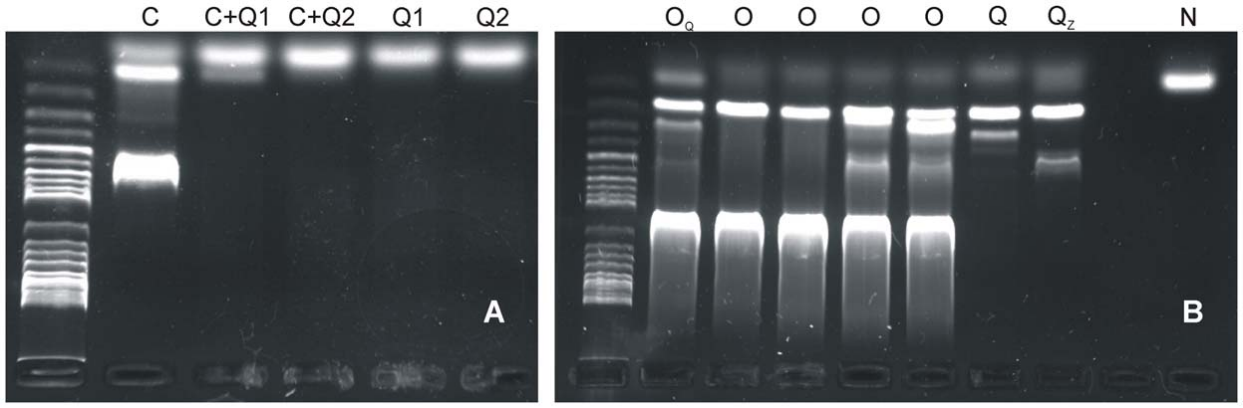
PCR results from extraction procedure trials. A: PCR for the COI gene on a positive control (C), two Atlantic *Diacavolinia* Qiagen extractions (Q1 and Q2) as well as reactions performed mixing 1 µL from the positive control and 1 µL from the same extractions as template. B: 18S gene amplification results from a Qiagen extraction (Q); Qiagen extraction after applying it through a Zymo column (Q_Z_); E.Z.N.A. extractions (O); and E.Z.N.A. extraction on an aliquot from a Qiagen extraction (O_Q_). N indicates a negative control. Only E.Z.N.A. extraction showed positive PCR results, suggesting the presence of inhibitors remaining when other kits were used. Ladder for both pictures: *ex*ACTG*ene* 1 kb Plus DNA ladder (Fisher).

PCR reactions carried out on individuals extracted with the Qiagen kit failed to amplify any product. Furthermore, the addition of 1 µl of extraction into a reaction containing a positive control (1 µL template) also resulted in failed amplifications, indicating the presence of PCR inhibitors on the extractions (Figure 2A). No improvement in success resulted from passing the extraction through the *OneStep™* PCR Inhibitor Removal Kit column (Fig. 2B). On the other hand, E.Z.N.A. extractions, from both new individuals and from aliquots from the previous Quiagen extractions, all resulted in successful amplifications (Fig. 2B). After extracting all individuals with the E.Z.N.A. kit, the final PCR success rate was >98% on the recently (2011) collected samples from the Atlantic Ocean. PCR success rate on the samples from the Pacific Ocean was lower (≈30%), indicating that not only fixation technique, but also storage conditions, are critical for molecular studies of this group of taxa. The findings of these tests serve as a reminder of the importance of optimizing methods for the particular chemistry of individual animal groups and detailed methodological descriptions. We recommend the use of this extraction method for further investigations on planktonic mollusks.

A 658 base pair (bp) region was used for the definitive multiple alignment and analyses, including sequences obtained from GenBank (the alignment file is provided in File S1). All the *Diacavolinia* and *Cavolinia* sequences encoded for the same amino-acid sequence with the exception of two positions (i.e., non-synonymous mutations): one exclusive to the *Diacavolinia* from the Pacific Ocean (a cysteine in contrast to serine from all the others at position 98); another found in all *Diacavolinia* from the Atlantic and some *C. uncinata* (valine instead of isoleucine). All sequences and specimen metadata can be found at the corresponding GenBank records (accession numbers JX183560-JX183616).

**Figure 3:**
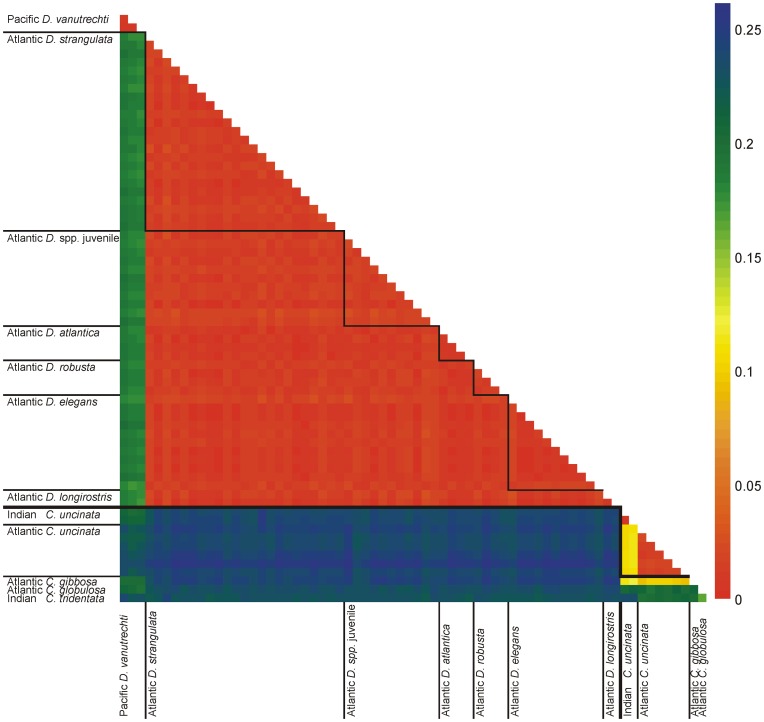
Heatmap of pair-wise K2P genetic distances. This heatmap depicts the genetic distances between each sampled individual based on the mitochondrial COI gene. Distances between specimens of the same species are outlined by black squares.

**Figure 4:**
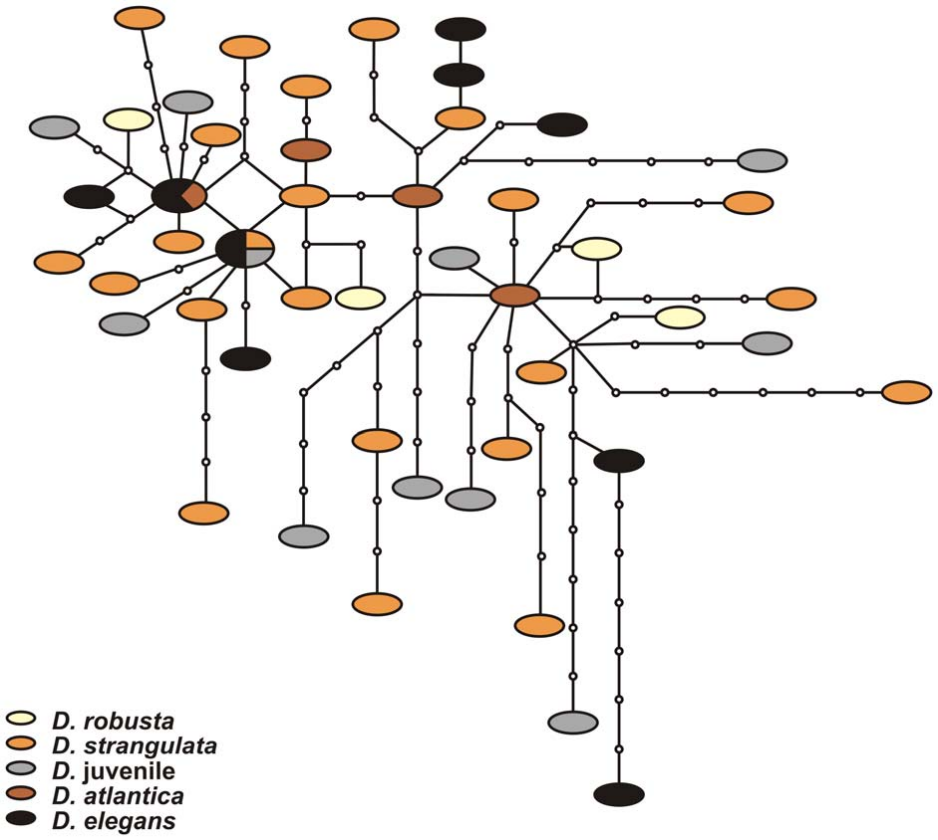
Haplotype network of Atlantic *Diacavolinia* species. This haplotype network depicts the variation within the COI region of the Atlantic *Diacavolinia* species. Each branch represents a mutational step, and small black circles depict the missing haplotypes needed to connect observed sequences. The size of the ovals is proportional to the number of sampled individuals with that haplotype. There is no species-related clustering among haplotypes.

All specimens from the Atlantic, despite distinct differences in shell morphology, showed genetic variation of <3% (K2P distance), with similar averages and ranges both within and between the different *Diacavolinia* species (Table 2; Figure 3). In some cases, no differences were observed between individuals from different morphospecies (the complete distance matrix file is provided in File S2). A significant deviation from neutrality was observed (Tajima’s *D* = −2.14, P<0.05), which could indicate an expanding population, purifying selection, or a low level of population structure [63], although this pattern is very common in marine animals and could also be explained by the hypothesis of sweepstakes reproductive success [64]. The Pacific and Atlantic samples were more distant (∼19%), a similar pattern to the observed differences when comparing *Cavolinia uncinata* from the Atlantic and Indian Ocean [∼11%, Figure 3; 26]. Based on these results, Pacific Ocean samples were excluded from the gene genealogy analysis where the TCS haplotype network diagram showed no discrimination between any of the species analyzed on the network, a high number of unique haplotypes (haplotype diversity, H_d_ = 0.993±0.006), and very few haplotypes shared by more than one individuals (Figure 4).

**Table 2:** Average, standard deviation, maximum and minimum K2P distances; within (w/i) the species *Diacavolinia elegans* (e.), *strangulata* (s.), juveniles/*aspina* (j.), *atlantica* (a.), and *robusta* (r.); between the five *Diacavolinia* spp. from the Atlantic (A.), within *Diacavolinia vanutrechti* from the Pacific (P.), between Atlantic and Pacific individuals of the *Diacavolinia,* and between all *Diacavolinia* and *Cavolinia* spp.

	w/i e.	w/i s.	w/i j.	w/i a.	w/i r.	Between A.	w/i P.	Atl./Pac.	*Diac*./*Cavo*.
Ave.	0.0131	0.0130	0.0157	0.0066	0.0103	0.0122	0.0082	0.1857	0.2384
StDv.	0.0038	0.0052	0.0051	0.0027	0.0065	0.0056	0.0009	0.0053	0.0088
Min.	0.0076	0.0015	0.0030	0.0046	0.0000	0.0000	0.0077	0.1735	0.1983
Max.	0.0170	0.0265	0.0265	0.0107	0.0233	0.0281	0.0092	0.2010	0.2617

**Figure 5:**
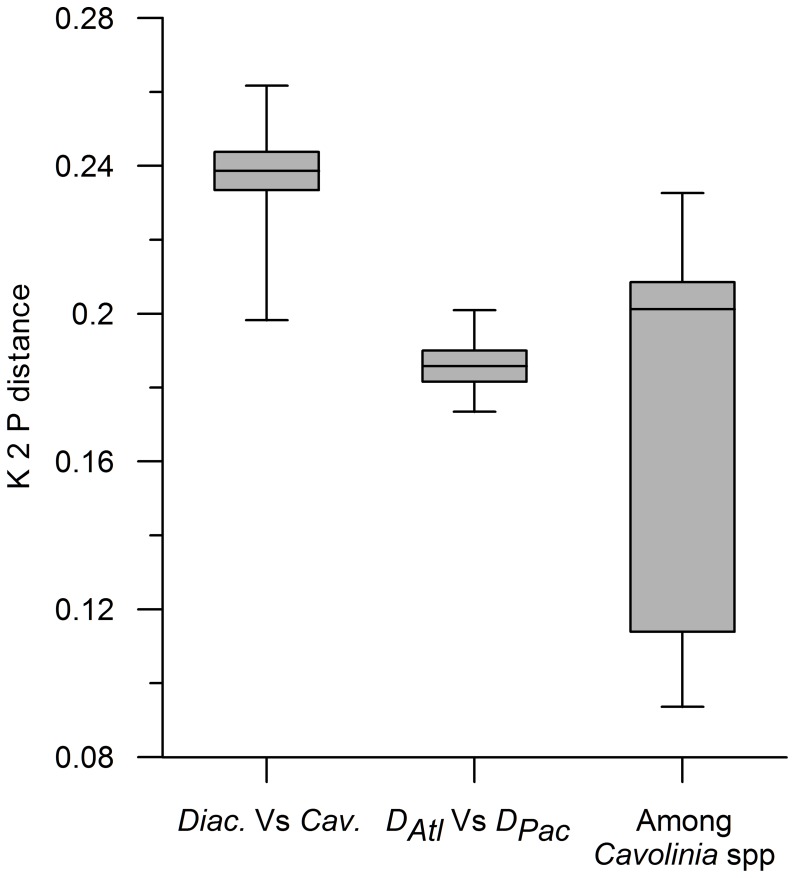
Distribution of the K2P distances between and within *Diacavolinia* and *Cavolinia* species. Box-Whisker plot showing the minimum, maximum, median, lower quartile, and upper quartile of K2P distances comparing *Diacavolinia* to *Cavolinia* spp., Atlantic to Pacific *Diacavolinia* individuals, and among *Cavolinia* species.

Comparisons of *Diacavolinia* spp. with *Cavolinia* spp. reveal larger distances (∼24%). Statistically, genetic distances showed a much higher average when comparing *Diacavolinia* spp. to *Cavolinia* spp. than within each genus (p<0.001 in both comparisons; Table 2, Figure 5). Distances between species of *Cavolinia* were significantly higher than distances between the Atlantic and Pacific populations of *Diacavolinia* (p<0.01; Figure 5).

**Figure 6:**
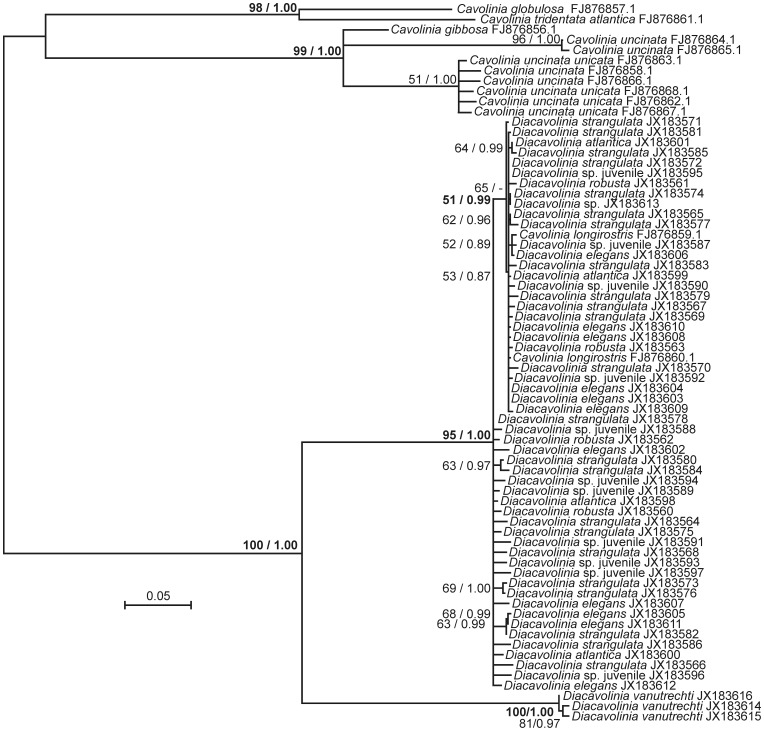
Tree analysis of the *Diacavolinia* spp. examined in this study and Cavoliniid sequences retrieved from GenBank. Topology and branch lengths reflect the tree with the best score under the Maximum Likelihood criteria (in RAxML). Numbers at nodes indicate the percentage bootstrap recovery and the Bayesian Posterior Probability of that node. All *Diacavolinia* from the Atlantic Ocean were recovered in a monophyletic clade, with no morphology–related subdivisions. *Diacavolinia vanutrechti* were shown as monophyletic, and distinct to the *Diacavolinia* from the Atlantic.

Trees showed consistency between both the Maximum Likelihood and Bayesian Inference methods, revealing the polyphyly of the Atlantic morphologically defined species(Figure 6). All *Diacavolinia* individuals from the Atlantic (both from this study and from GenBank) were clustered in a single node, without any morphology-related pattern of discrimination within the clade. Some smaller sub-clades of these sequences were statistically supported, but these often contained individuals belonging to different *Diacavolinia* species. At a genus level, *Diacavolinia* appeared as monophyletic: the Pacific Ocean individuals, identified via the morphological key as *D. vanutrechti*, clustered in a monophyletic sister clade to the Atlantic *Diacavolinia* spp. Very low distances were evident within the Atlantic and Pacific clades, whereas much longer branches connected the two nodes. In our tree analyses *Diacavolinia* spp. were a significantly distinct branch of the Cavoliniinae. The rest of the subfamily cluster showed *Cavolinia globulosa* and *Cavolinia tridentata* associated together in a separate clade from *Cavolinia uncinata*. *Cavolinia uncinata* were represented by two distinct groupings and were closely associated with *Cavolinia gibbosa*.

## Discussion

Our results suggest that the designation of these forms by van der Spoel et al. [31] as separate species is likely inappropriate. Despite distinct differences in shell morphology that were the basis of the original species descriptions, there were no significant differences between the four different morphological forms of Atlantic *Diacavolinia* studied in both tree and distance-based analyses. There are differences, however, in *Diacavolinia* spp. on a basin scale with an approximately ten times higher distance in the between-basin, also shown in the tree analyses, consistent with the presence of a species-related barcode gap [41], [65]. The differences between the Pacific and Atlantic *Diacavolinia* sequences, suggestive of species level distinctions between populations, are similar to those previously reported between congeneric species in pteropods [26], although high differences within the same species between ocean basins have been reported for many holoplanktonic taxa [26], [66], [67], [68], [69]. Overall, these findings indicate that some of the species identifications established by van der Spoel et al. [31] should be reconsidered, although any renaming and reclassifying of species will need to await the collection of more samples from a broader geographic distribution for genetic analysis of all of van der Spoel’s 22 putative species. In contrast, the high genetic distances between the genera *Diacavolinia* and *Cavolinia* as well as the unique reproductive structures and locking mechanism described by van der Spoel [31], would support the persistence of *Diacavolinia* as a separate genus. For the *Cavolinia* species retrieved from GenBank for comparison and framing the genera, our results were consistent with those obtained and discussed by Jennings et al. [26].

Recent tests of the effect of spatial and numerical coverage on barcode efficacy for resolving patterns in species identification emphasize the importance of large sample size (ranging from at least 150-50 individuals per species) and an awareness of changes in the ability of software to recognize a barcode gap at larger geographic scales [70], [71], [72]. As our *Diacavolinia* sampling was only at four locations, three in a relatively constrained region of the NW Atlantic Ocean, and since we had a somewhat limited number of individuals, particularly in the Pacific, it is likely that further sampling would change the fine details of the pattern we have documented. The pronounced barcode gap would probably decrease in size with increased distributional coverage and sample size, and the diversity in the Pacific group would increase. However, the genetic distances between the Atlantic and Pacific populations of *Diacavolinia* are larger than most previously documented populations from the same planktonic species [26], [66], [71], even those that exist in multiple ocean basins. We feel, therefore, that greater sampling would not change our central conclusions and our findings are minimally suggestive that existing species delimitations should be re-visited.

The differences in shell morphology within the Atlantic *Diacavolinia*, the monophyletic recovery of all the individuals without segregation among the different species, and the low K2P distances in their COI sequence (<3%) suggest a high level of phenotypic variation in regard to shell morphology within the species. Although in their various studies, van der Spoel and colleagues frequently found sympatry of shell morphologies, it was concluded that these differences were a result of genetic variation. Alternate explanations include a shell form which is not under strong selective pressure or a shell form that is influenced by differences in life history and developmental environment, as was previously suggested by McGowan [40]. One clue to the possible mechanism by which the shell shape of these animals could be influenced by environment involves the development of the adult teloconch in the Cavoliniinae subfamily. Although often overlooked, it has been documented in the literature that individuals of this group develop their adult shell in a unique way. The adult teloconch is rapidly extended from the somewhat flattened and curved conical juvenile protoconch. When the teloconch reaches a large size the growing animal extrudes its mantle to surround the shell, decalcifies the structure and then rapidly mechanically increases the shell volume and recalcifies the shell in the characteristically bulging adult shape, then loses the juvenile protoconch [73]. All further growth is predominantly in the soft parts, although extensions of the rostrum and lateral spines or thickening of the shell may continue throughout life, a fact that may result in individuals with similar length shells containing animals of varying age, mantle size, and overall mass. This relationship between overall mass and shell length was found by Gilmer [28], although the mechanism at that time was not discussed. This demineralization process has also been documented in *Cavolinia uncinata* and may produce a similar variation in shell morphology. If disparities in shell shape, which have been used to designate forma in *Cavolinia uncinata*, are similarly exclusively a result of developmental variation, this could explain the lack of genetic support for their differentiation found in Jennings et al. [26]. The decalcification and recalcification of the shell, and the energetic investment available for the deformation may be dependent on food availability, physiological state, water temperature and environmental carbonate chemistry. Variations in any of these factors might result in profound differences in shell angle and size, which could explain the sympatry of shell forms. This developmental mechanism bears further investigation, particularly as the carbonate chemistry of the ocean changes in response to anthropogenic forcing. The sensitivity of this group of thecosomatous pteropods to ocean acidification may be impacted by this phenotypic variation and their control over calcification.

Our results have a number of implications for biodiversity, ecology, and population resiliency in the face of changing oceanic conditions. The species level differences between *Diacavolinia* from the Pacific and Atlantic Oceans in this study are similar to the regional differences in other Cavolinid species, including *Cavolinia uncinata, Cuvierina columnella, Diacria major,* and *Clio cuspidata* documented by Jennings et al. [26]. These findings suggest that further investigations of Indian Ocean, Red Sea and Southern Hemisphere populations of *Diacavolinia spp.* may reveal a number of regional species. It is important to investigate such groups to determine their taxonomic differences, as there may be functional variability in the populations. This is particularly important with regards to studies of their sensitivity to changing environmental conditions, as there are ocean basin differences in hydrography that may result in different natural exposures and subsequently different physiological sensitivities to stressful conditions. For example, individuals of the Pacific population of *Diacavolinia*, although they were identified as *Cavolinia longirostris* in previous studies, are known to undergo a diel vertical migration into an oxygen minimum zone, where the water is thought to be undersaturated with respect to aragonite, pH is ∼7.5, and oxygen is well below 20 µM O_2_ kg^−1^ [74], [75], [76]. This oxygen minimum/carbon maximum zone is extensive in the Pacific Ocean, whereas similar conditions are absent from the Atlantic [74]. Individuals of *Diacavolinia* from the Pacific were not responsive to short term laboratory exposure to conditions of elevated carbon dioxide (∼1000 ppm); however, since this population is genetically distinct from the Atlantic, as indicated by this study, then it is possible that their physiological responses may be different as well, and the Atlantic individuals could be more sensitive to enhanced carbon dioxide. As an indicator, the single mutation that is exclusive to the Pacific *Diacavolinia* (cysteine instead of serine) might not be trivial, since cysteines are the ligand points for the copper units in the cytochrome *c* oxidase complex [77], and they also are known to be regulation points in other oxidases [78], [79]. Subsequently, this difference in the COI sequence may influence the efficiency of the respiratory chain and therefore the sensitivity of these populations of animals to oxygen concentration and internal pH. Having accurate identifications of species, together with an understanding of their basin scale distribution and biodiversity, could therefore be critical for making informed estimates of planktonic ecosystem sensitivity to anthropogenic change.

## Supporting Information

File S1 File containing the aligned 658 base pair (bp) COI region for Cavoliniinae from this study and from Genbank.
(FAS)

File S2 Distance matrix (K2P) for the Cavoliniinae from this study and from Genbank.
(XLSX)
